# A meta-analysis on the effect of operation modes on the recurrence of papillary thyroid microcarcinoma

**DOI:** 10.18632/oncotarget.12698

**Published:** 2016-10-15

**Authors:** Dandan Yi, Peng Song, Tao Huang, Xiaoqiao Tang, Jianfeng Sang

**Affiliations:** ^1^ Department of general surgery, Drum Tower Clinical Medical College of Nanjing Medical University, Nanjing, Jiangsu Province, China; ^2^ Department of General Surgery, Nanjing Drum Tower Hospital, Nanjing, Jiangsu Province, China

**Keywords:** papillary thyroid microcarcinoma (PTMC), recurrence rate, total thyroidectomy, non-total thyroidectomy, meta-analysis

## Abstract

Whether total thyroidectomy reduces the recurrence rate in patients with papillary thyroid microcarcinoma (PTMC) is currently controversy. Conclusions of sporadic, inconsistent, and mono-institutional studies need a meta-analysis to evaluate. 525 relevant studies were obtained from initial search on PubMed, 511 studies were excluded by inclusion and exclusion criteria. Eligible data were extracted from each included study. The Odds ratios (ORs) and 95% confidence interval (CI) were used to assess the difference in the recurrence rates between PTMC patients treated with total thyroidectomy and non-total thyroidectomy. OR and 95% CI were calculated using a fixed-effects or a random-effects model. The Q statistic was used to evaluate homogeneity and Beggs test was used to assess publication bias. 14 studies meeting the inclusion criteria were included in this meta-analysis. The over all recurrence rates of pooled patients with total thyroidectomy and non-total thyroidectomy were 2.83% and 2.84% respectively. Primary random-effects model analysis showed, no significant difference of recurrence rates existed between two operation modes (OR = 0.732, 95% CI: 0.444 - 1.208), while, high heterogeneity among studies was found, I-squared index (I2) = 40.2%. After remove one study with high heterogeneity, the OR of the pooled recurrence rates of the total thyroidectomy and the non-total thyroidectomy groups was 0.786 (95% CI: 0.363 - 1.701), further suggesting no significant difference of the recurrence rate exists between two operation modes. Our meta-analysis demonstrated postoperative recurrence of PTMC is not reduced by total thyroidectomy, non-total thyroidectomy is also a good choice to treat PTMC patients.

## INTRODUCTION

Papillary thyroid cancer (PTC) is the most common type of thyroid cancer making up to -80% of all thyroid cancer cases [1]. PTC can occur at any age, and its incidence has been increasing over the last decades [1]. Papillary thyroid microcarcinoma (PTMC) is a subgroup of PTC, which refers to the tumor diameter is or less than 1cm in PTC according to the World Health Organization definition [1]. The incidence of PTMC increased year by year and reached as high as 11.8% of the total population in 2008 [2]. PTMCs are most often slow growing tumors and most can be removed surgically [2–5]. Although slow-growing PTMC can sometimes spread to the lymph nodes in the neck, positive lymph nodes do not usually worsen the generally excellent prognosis [2–5]. The involved lymph nodes can be surgically removed along with the thyroid, thus, most people diagnosed with PTMC will not die from it [2–5]. Nevertheless, the surgical treatment of PTC is controversial. Some scholars believe that although the prognosis of PTMC is good, there is still a risk of recurrence and metastasis. So, these scholars advocate total thyroidectomy, that is, lobectomy of whole thyroid, including right lobe, left lobe, pyramidal lobe and isthmus, and even central lymph node dissection when necessary [6–10]. On the contrary, some other scholars believe that patients with PTMC underwent total thyroidectomy belongs to excessive treatment [11], because no sufficient evidence to show total thyroidectomy can reduce the risk of recurrence and mortality; subtotal thyroidectomy can achieve the therapeutic effect [12,13] and reduce the incidence of complications of total thyroidectomy [14]. In addition, a study suggests that, although PTMC in young patients may be more progressive than in older patients, it might not be too late to perform surgery after subclinical PTMC has progressed to clinical disease, regardless of patient age [15]. Obviously, although current consensus believes that PTMC prognosis is generally excellent, there is controversy on the operation mode of PTMC patients. Existing controversy lacks verification of multi-center study with large sample size. Therefore, we summarized operation mode and recurrence of the previous studies on PTMC using a meta-analysis. Our report will be helpful for the correct treatment of PTMC.

## RESULTS

### Search results

**Figure 1:**
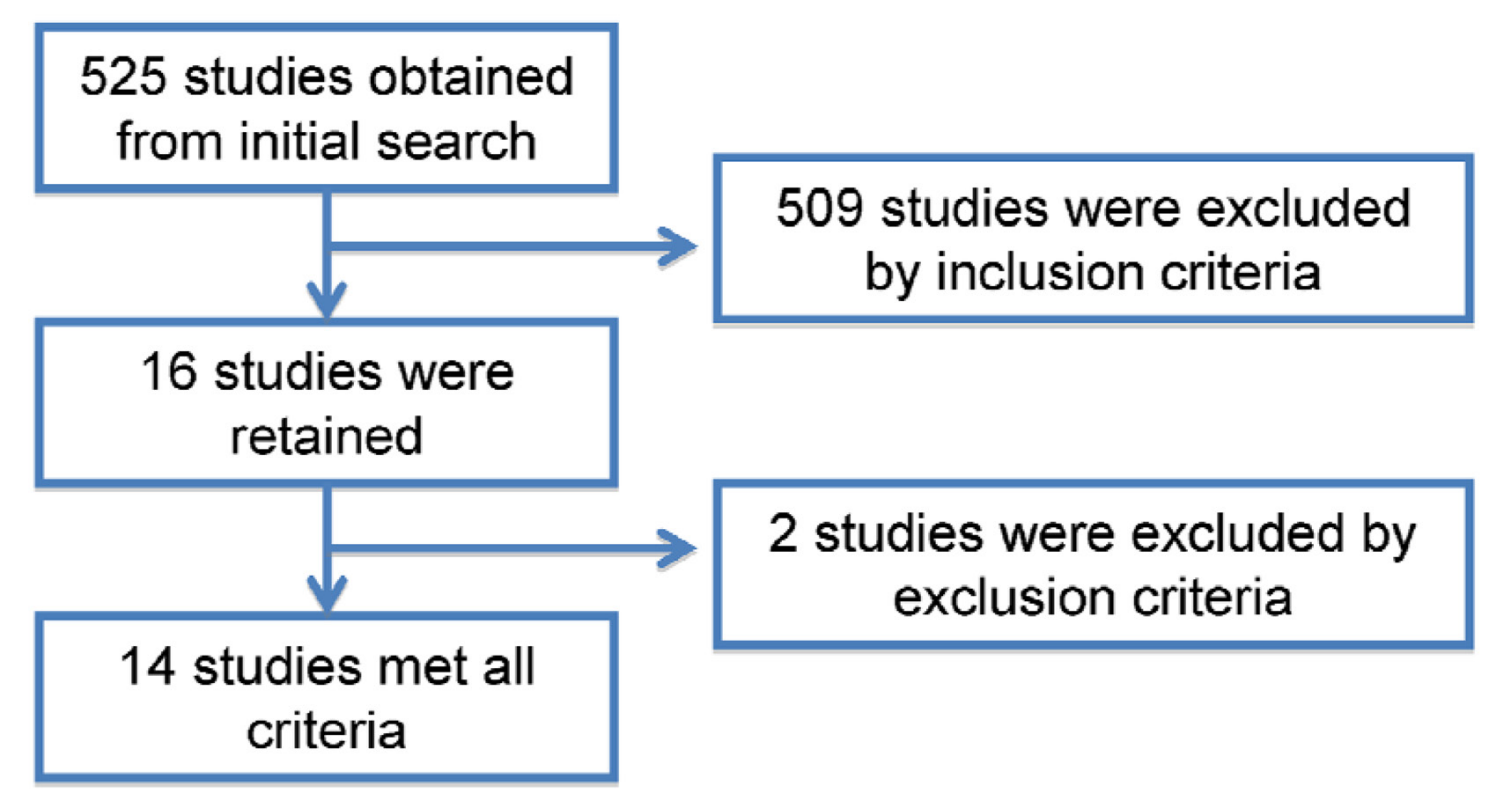
Flow chart of literature search and study selection 14 studies meeting the inclusion criteria were included in this meta-analysis.

525 articles were initially identified in PubMed. After review of the titles and abstracts, 509 studies were excluded. Whereas, 16 were considered to be potentially relevant. Of the 16 studies, the “total thyroidectomy” of two studies was defined as total thyroidectomy and near total lobectomy, which did not meet our definition of total thyroidectomy and then were next excluded. Finally, 14 studies meeting the inclusion criteria were included in this meta-analysis (Figure 1 and Table 1) [16–29].

**Table 1:** Summary of fourteen included studies

Original report	Year	Total subjects	Total thyroidectomy		Non-total thyroidectomy		P
			N	Recurrence	N	Recurrence	
Cho et al [16]	2015	336	182	8 (4.4%)	154	8 (5.2%)	0.463
Lee et al [17]	2014	2018	1245	26 (2.1%)	773	15 (1.9%)	0.823
Mantinan et al [18]	2012	91	77	7 (9.1%)	14	1 (7.1%)	1.000
Pelizzo et al [19]	2006	403	359	1 (0.3%)	44	5 (11.4%)	<0.001
Kuo et al [20]	2011	61	52	6 (11.5%)	9	0 (0%)	0.367
Caliskan et al [21]	2012	842	428	7 (1.6%)	414	12 (2.9%)	0.158
Kim et al [22]	2015	1661	1140	18 (1.6%)	521	9 (1.7%)	0.823
Ardito et al [23]	2012	149	135	28 (20.7%)	14	0 (0%)	0.047
Jin-Kyu Cho [24]	2012	527	294	9 (3.1%)	233	8 (3.4%)	0.499
Pelizzo et al [25]	2004	149	126	0 (0%)	23	3 (13.0%)	0.003
Pedrazzini et al [26]	2013	231	177	10 (5.6%)	54	5 (9.3%)	0.256
Saaduddin [27]	2016	184	148	5 (3.4%)	36	0 (0%)	0.343
Gülben et al [28]	2008	81	64	1 (1.6%)	17	0 (0%)	0.790
Appetecchia et al [29]	2002	106	92	2 (2.2%))	14	0 (0%)	0.756
Total		6839	4519	128 (2.8%)	2320	66 (2.8%)	1.000

Total thyroidectomy was defined as lobectomy of whole thyroid with or without neck lymph node dissection; non-total thyroidectomy includes near total/subtotal thyroidectomy, lobectomy or lobo-isthmectomy regardless of lymph node dissection. The data of recurrence were presented as number (percentage). One-tailed P of Fisher exact probability test was presented. N, number of subjects in each group

### Characteristics of the studies

All fourteen eligible studies were published between 2002 and 2016. Five studies were conducted in the Republic of Korea [16, 17, 21, 22, 24], one in Spain [18], five in Italy [19, 23, 25, 26, 29], one in Taiwan [20], one in United State of American [27], and one in Turkey [28]. Thirteen reports were retrospective studies [19–25, 27–29], and one was prospective study [26]. The follow up durations for the recurrence of PTMC range from 2 to 24 years and the study sample sizes range from 81 to 2018 (Table 1). The fourteen studies showed inconsistent results regarding PTMC recurrence rate between patients with total thyroidectomy and patients with non-total thyroidectomy. 11 out of the 14 studies showed no significant difference of the recurrence rate between two operation groups; two studies showed that the recurrence rates were significantly higher in patients treated with non-total thyroidectomy [19, 25]; only one study showed that the recurrence rate in total thyroidectomy group was significantly higher than that in non-total thyroidectomy group [23]. These fourteen studies assembled 6839 patients in total, 4519 and 2320 were treated with total thyroidectomy and non-total thyroidectomy, respectively. The over all recurrence rates for patients with total thyroidectomy and non-total thyroidectomy were 2.83% and 2.84% respectively, no statistical difference between the two groups was observed (Table 1).

### Pooled analysis on recurrence rate

**Figure 2:**
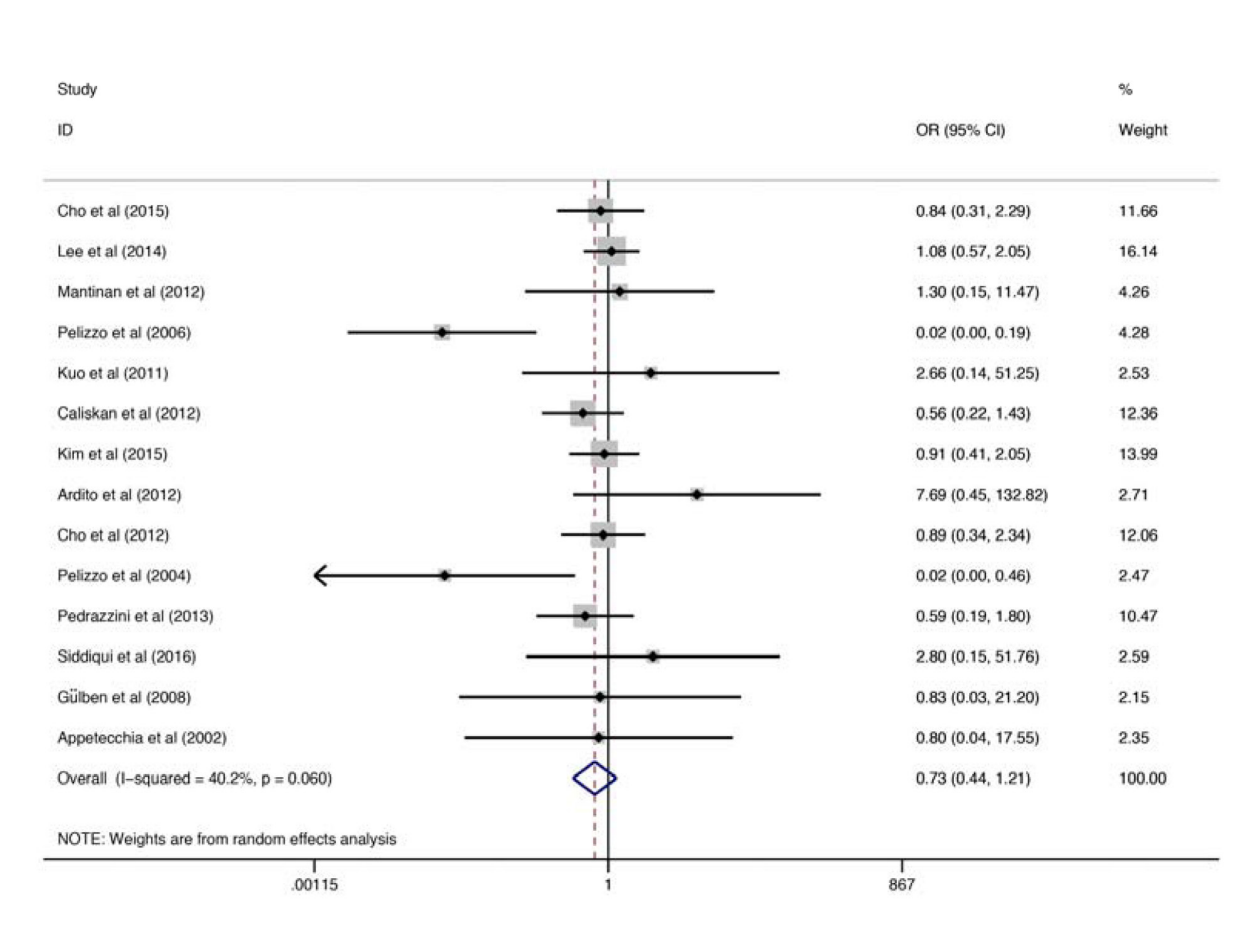
Forest plots for the recurrence rates The squares correspond to the study specific OR and 95% CI. The area of the squares reflects the weight. The diamond represents the summary OR and 95% CI. OR, odds ratio; CI, confidence intervals.

The original data of study year, study sample size as well as the recurrence rate were summarized in Table 1. Random-effects model analysis showed that the pooled estimate (OR) of difference in the recurrence rate between the total thyroidectomy and the non-total thyroidectomy groups was 0.732 [95% CI, 0.444 to 1.208; P = 0.222] (Figure 2). Although above pooled analysis showed that there is no significant difference in the recurrence rate between two groups, the I2 and the p value of the Q test were 40.2% and 0.060 respectively, suggesting significant heterogeneity among the studies.

### Heterogeneity and subgroup analyses

**Figure 3:**
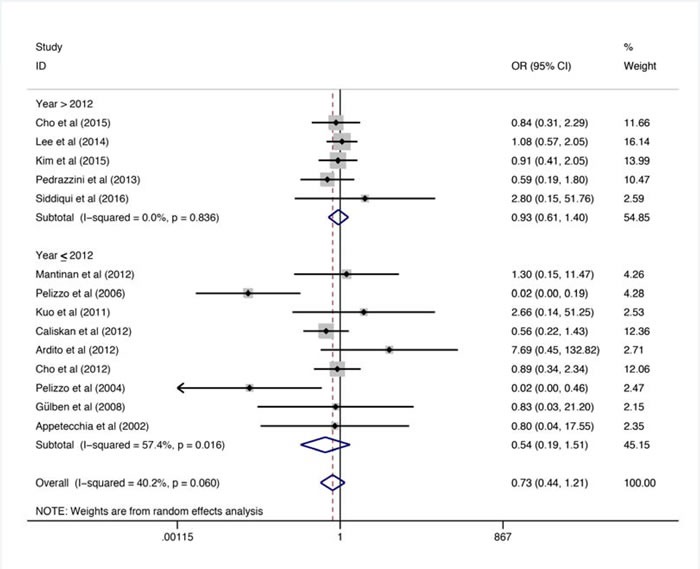
Subgroup Forest plots for the recurrence rates The squares correspond to the study specific OR and 95% CI. The area of the squares reflects the weight. The diamond represents the summary OR and 95% CI. OR, odds ratio; CI, confidence intervals.

**Figure 4:**
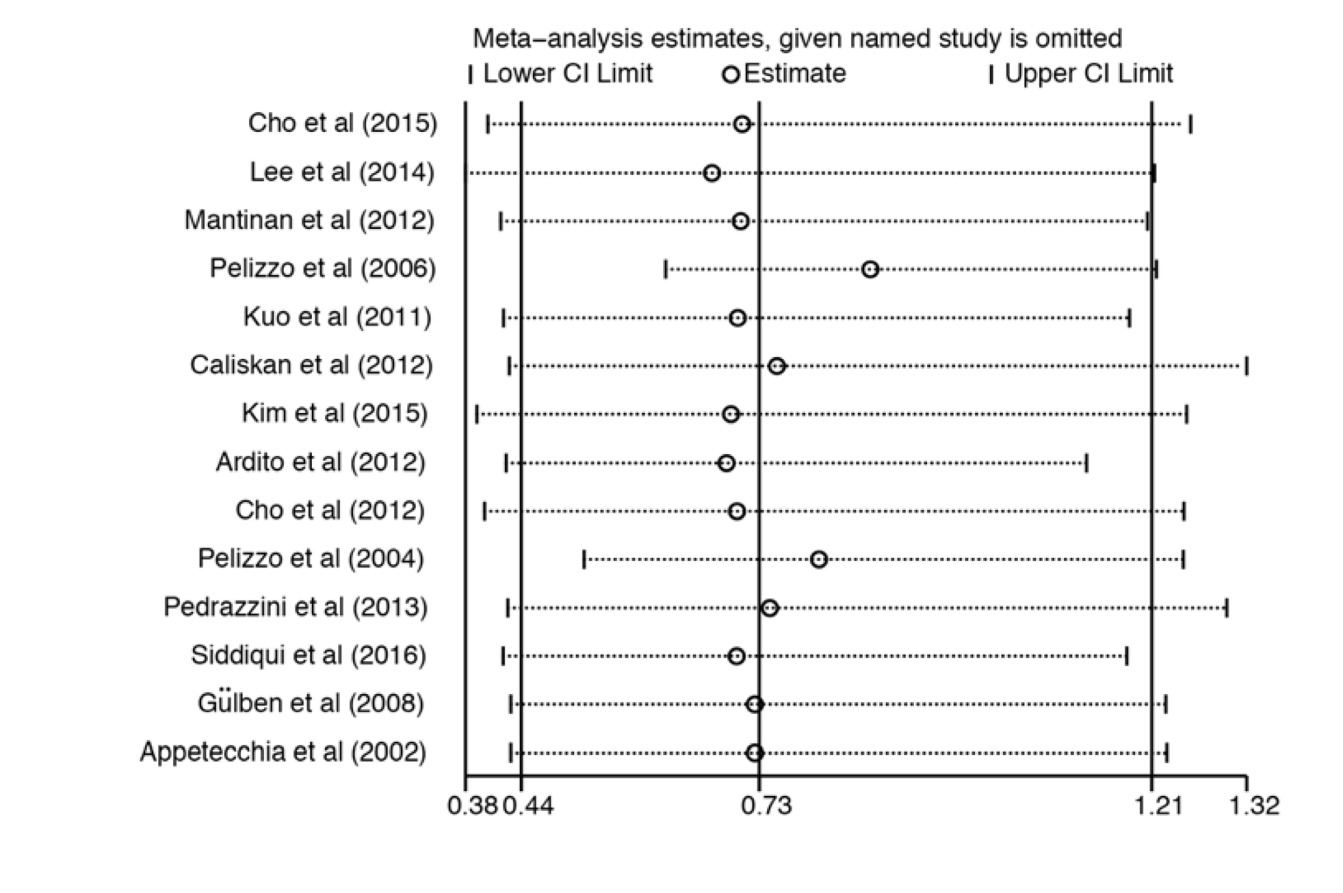
Sensitivity analyses The OR and 95% CI of each study. OR, odds ratio; CI, confidence intervals.

Subgroup meta-analysis showed that, the I2 and the p value of the Q test of studies performed in and before 2012 were 57.4 % and 0.016 respectively; and the I2 and the p value of the Q test of studies performed after 2012 were 0.0 % and 0.836 respectively (Figure 3). Although also no significant difference was observed in the recurrence rates between two groups (total thyroidectomy and non-total thyroidectomy groups) both in pooled studies performed in and before 2012 (OR = 0.539, 95% CI: 0.192 - 1.508) and studies performed after 2012 (OR = 0.928, 95% CI: 0.614 - 1.403), the subgroup analysis indicates that the heterogeneity came from the studies before 2012. Further sensitivity analysis revealed that heterogeneity mainly caused by one study performed by Pelizzo et al [19] (Figure 4). When above study was removed from the model, the I2 and the p value of the Q test of studies performed in and before 2012 were 24.2 % and 0.236 respectively, suggesting the heterogeneity was reduced obviously. As expected, after remove the heterogeneous study, the estimate (OR) of difference in the recurrence rate between the total thyroidectomy and the non-total thyroidectomy groups was 0.866 (0.619-1.213), further suggesting no significant difference of the recurrence rate exist between two operation modes.

### Publication Bias

**Figure 5:**
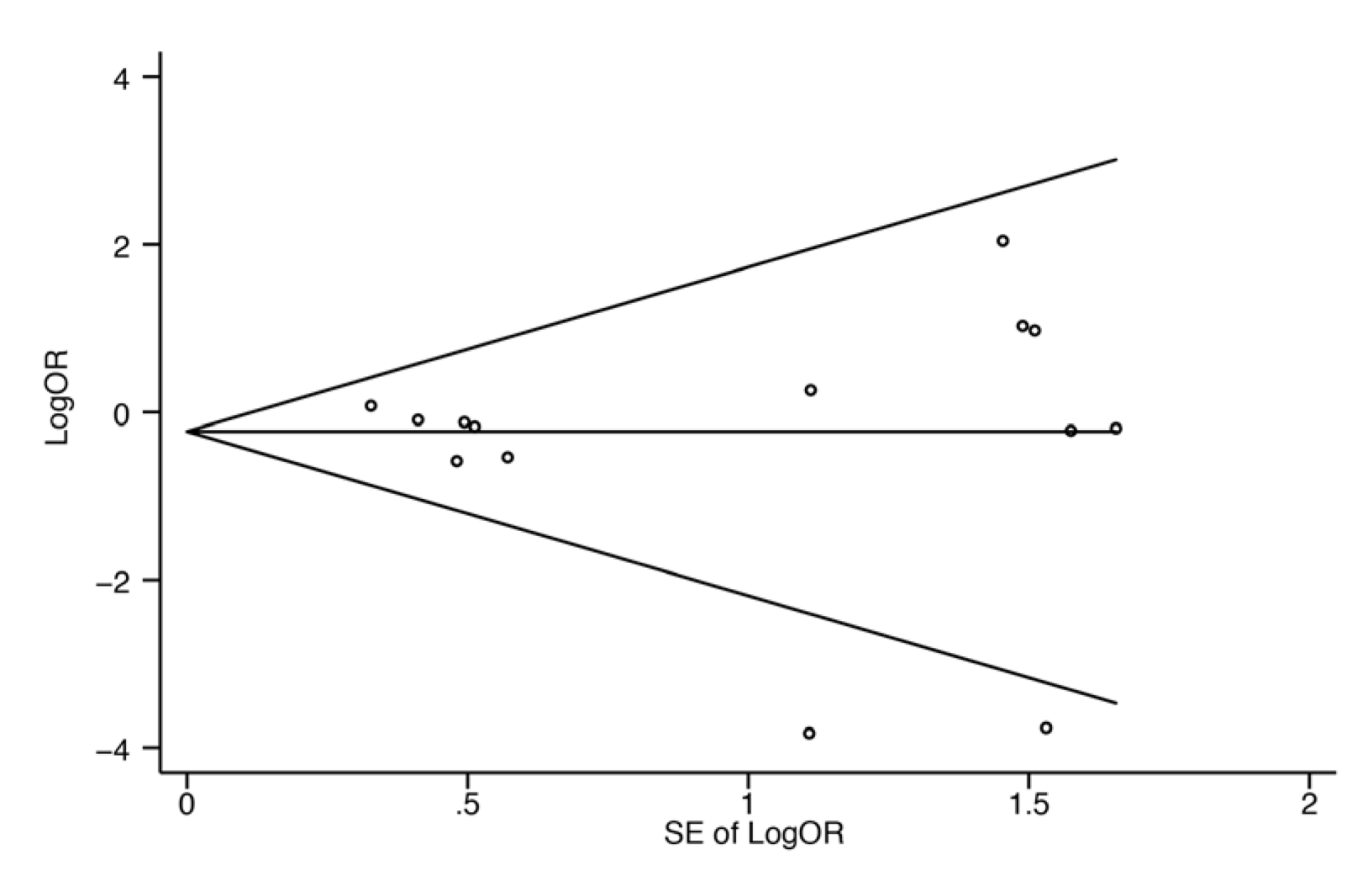
Begg's funnel plot with pseudo 95% confidence limits Each circle represents a separate study for the indicated association. Log [OR], natural logarithm of odds ratio. SE, standard error.

Begg's test was used to evaluate publication bias in the literatures. The shapes of the funnel plots revealed no obvious asymmetry (Figure 5). We then used Begg's test to assess statistical evidence of funnel plot symmetry, but no significant publication bias was indicated (Pr > |Z| = 0.622).

## DISCUSSION

PTC is the most common tumor of thyroid account for 80% of all malignant tumor of thyroid [30]. WHO defined PTC less than 1 cm in diameter as PTMC, which is the most common type of PTC [31]. PTMC is highly prevalent worldwide; fortunately, the prognosis of PTMC is good; the recurrence rates of 20 and 40 years were 6% and 8% respectively [3]. The mortality is also very low, more than 99% patients did not occur distant metastasis or die from PTMC [8]. A study with 18,445 PTMC patients showed that, the over all survival rates of 10 and 15 years were 94.6% and 90.7% respectively and disease-specific survival rates were 99.5% and 99.3% respectively [32]. Appetecchia et al and Lin et al's studies also showed the good prognosis of PTMC after surgery and regardless of total thyroidectomy or non-total thyroidectomy [29, 33]. Nonetheless, It is controversial regarding whether or not total thyroidectomy and prophylactic cervical lymph node dissection should be performed in patients with PTMC [6–13, 34, 35]. In addition, some scholars believe that the treatment should not be different between patients with PTC and patients with PTMC [31, 36]. Sporadic, regional and mono-institutional reports can't provide a convincing conclusion. Therefore, a meta-analysis focused on how to treat the PTMC patients is urgent needed for clinicians. In this report, the over all recurrence rates for pooled patients with total thyroidectomy and non-total thyroidectomy were 2.83% and 2.84% respectively, no difference of recurrence rates was found between two operation modes. Further heterogeneity and subgroup analyses showed that, one study [19], showing significantly higher recurrence rate in patients treated with non-total thyroidectomy, has a high heterogeneity. After remove this study, the estimate (OR) of difference in the recurrence rate between the total thyroidectomy and the non-total thyroidectomy groups was 0.866 (0.619-1.213), further suggesting no significant difference of the recurrence rate exist between two operation modes.

The recurrence of PTMC correlated with its pathological characteristics as well as other factors: gender, age, tumor size, multifocal carcinoma, extrathyroidal extension, capsular infiltration, lymph node metastasis as well as radiation therapy [37]. These issues could not be addressed in one single meta-analysis due to lack of integrity of the original studies, no available original study had addressed all above issues entirety. In spite of this, there are studies addressed these issues partially from different perspectives. Studies showed that the recurrence rate in patients with lymph node metastasis is higher than that of no lymph node metastasis and lymph node metastasis is an independent prognostic factor in patients with [38–40]. Due to the effect of lymph node metastasis on recurrence and prognosis, some scholars suggested lymph node dissection for treatment of patients with lymph node enlargement [41, 42]. Some studies believe that total thyroidectomy is not necessary for all PTMC patients; extrathyroidal invasion and cervical lymph node metastasis is highly invasive, patients with these kind of PTMCs were easy to recurrence and poor prognosis; thus, total thyroidectomy is recommended [16, 43, 44]. In patients with only one or no cervical lymph node metastasis, lobectomy or subtotal thyroidectomy can be selected [17]. The incidence of postoperative complications can be reduced by non-total thyroidectomy [14]. In addition Mantinan et al's study showed that, multifocal tumor is a prognostic factor, tumor size and postoperative radiation therapy did not associated with prognosis [18]. So et al's study suggested total thyroidectomy for patients with multifocal PTMC [45], and postoperative radiation treatment did not reduce the risk of 10-years-recurrence and PTMC related death. However, some researchers have different opinions. Lee et al found no association between lymph node metastasis and recurrence [47]. Kim et al believed that, the multifocal or single focal tumors did not affect the recurrence, multifocal tumors associated with prognosis when the tumor size is larger more than 1 cm in diameter, while, multifocal tumors had no effect on the prognosis in patients with PTMC [22]. Again, sporadic, regional and mono-institutional reports can't provide a convincing conclusion, more works are need to figure out all above issues.

There are many defects in our research. First, retrospective studies have inherent defects such as selection bias and inaccurate. Secondly, we mainly studied the recurrence rates of total thyroidectomy and non-total thyroidectomy; we had not studied the recurrent differences between total thyroidectomy, near-total/subtotal thyroidectomy, lobectomy and lobo-isthmectomy.

## CONCLUSION

Postoperative recurrence of PTMC is not related to operation modes (total thyroidectomy or non-total thyroidectomy), so, non-total thyroidectomy is also a good choice to treat PTMC patients.

## MATERIALS AND METHODS

This study was strictly reported in accordance PRISMA (Preferred Reporting Items for Systematic Reviews and Meta-Analyses) Guidelines.

### Search strategy, study selection and data extraction

To identify all eligible studies that address the recurrence rate of PTMC treated with total thyroidectomy or non-total thyroidectomy, we search the available literatures in the PubMed using the following terms: papillary thyroid microcarcinoma and recurrence, PMC and recurrence, PTMC and recurrence, papillary thyroid microcarcinoma and prognosis, PMC and prognosis or PTMC and prognosis. The final search was updated in June 2016. The search was limited to studies published in English. Two investigators performed the data search, data screening, and data extraction independently. Disagreements regarding data screening and extraction were resolved by discussion. If there was still disagreement, a third reviewer participated to resolve the issue. The exclusion and inclusion criteria were as follows: 1) published documents, excluding the review; 2) research objects are PTMC patients; 3) studies using total thyroidectomy or non-total thyroidectomy and have recurrence data; 4) studies with subjects greater than 50, postoperative follow-up were performed in all involved subjects, P value was set at 0.05.

Total thyroidectomy was defined as lobectomy of whole thyroid (including right lobe, left lobe, pyramidal lobe and isthmus) with or without neck lymph node dissection; non-total thyroidectomy includes near total/subtotal thyroidectomy, lobectomy or lobo-isthmectomy regardless of lymph node dissection. The following data of each study will be extracted: authors of the study; time of publication; sample size; number of recurrent events after total thyroidectomy or non-total thyroidectomy; number of patients without recurrence after total thyroidectomy or non-total thyroidectomy.

### Data synthesis and statistical analyses

Meta-analysis was performed using the Stata software version 12.0 (StataCorp LP, College Station, TX). Considering the differences in patient selection, operation mode and definitions of observation among studies; and these differences might cause heterogeneity in following meta-analysis; fixed-effect and random effects models were adopted to merge the data. The heterogeneity across studies was detected by Cochrane Q test (χ2 test) and I-squared index (I2). I2 = 0% ~ 25% indicates no heterogeneity among the studies; I2 = 25% ~ 50% indicates low heterogeneity among studies; I2 = 50% ~ 75% indicates moderate heterogeneity among studies; I2 = 75% ~ 100% indicates significant heterogeneity among studies. If an existence of statistical heterogeneity was observed, the data were analyzed using a random-effects model. Otherwise, the data were considered to be homogeneous and a fixed model was employed. Odds ratio (OR) and corresponding 95% confidence intervals (CIs) was adopted in the assessment of differences in means of recurrence rate. α was set at < 0.05.
